# Serial analysis of cytokine and chemokine profiles and viral load in severe fever with thrombocytopenia syndrome: Case report and review of literature

**DOI:** 10.1097/MD.0000000000017571

**Published:** 2019-10-18

**Authors:** Keita Fujikawa, Tomohiro Koga, Takahide Honda, Toshihisa Uchida, Momoko Okamoto, Yushiro Endo, Tomo Mihara, Akira Kondo, Satoshi Shimada, Daisuke Hayasaka, Kouichi Morita, Akinari Mizokami, Atsushi Kawakami

**Affiliations:** a Department of Rheumatology, Japan Community Healthcare Organization, Isahaya General Hospital, Eishohigashi-machi, Isahaya; b Department of Immunology and Rheumatology, Graduate School of Biomedical Sciences, Nagasaki University, Sakamoto, Nagasaki; c Department of Internal Medicine; d Department of Infectious Disease Medicine, Japan Community Healthcare Organization, Isahaya General Hospital, Eishohigashi-machi, Isahaya; e Department of Virology, Institute of Tropical Medicine, Nagasaki University, Sakamoto, Nagasaki, Japan.

**Keywords:** chemokine, cytokine, encephalopathy, severe fever with thrombocytopenia syndrome, viral load

## Abstract

**Rationale:**

Severe fever with thrombocytopenia syndrome (SFTS) is a recently recognized fatal infectious disease caused by the SFTS virus, and severe cases are complicated by the presence of hemophagocytic lymphohistiocytosis (HLH) associated with a cytokine storm. Herein, we report on serial changes of serum cytokine levels and viral load in a severe case of SFTS.

**Patient concerns:**

A 63-year-old Japanese woman presented with high-grade fever, abdominal pain, diarrhea, impaired consciousness, leukocytopenia, and thrombocytopenia.

**Diagnosis:**

SFTS was diagnosed based on a positive serum test for SFTS virus RNA and electroencephalogram (EEG) findings of encephalopathy.

**Interventions:**

The patient was treated with supportive therapy, including steroid pulse therapy (intravenous methylprednisolone 1 g/d for 3 days) for HLH and intravenous recombinant thrombomodulin 19200 U/d for 7 days for disseminated intravascular coagulation.

**Outcomes:**

Treatment for 7 days improved both symptoms and abnormal EEG findings, and SFTS virus RNA disappeared from the serum at day 10 from the onset of symptoms. The serum cytokines and chemokines analysis during the clinical course revealed 2 distinct phases: the acute phase and the recovery phase. The cytokines and chemokines elevated in the acute phase included interleukin (IL)-6, IL-10, interferon (IFN)-α2, IFN-γ, tumor necrosis factor-α, interferon-γ-induced protein-10, and fractalkine, while the IL-1β, IL-12p40, IL-17, and vascular endothelial growth factor levels were higher in the recovery phase.

**Conclusion:**

These observations suggest that the cytokines and chemokines elevated in the acute phase may reflect the disease severity resulted in a cytokine storm, while those in the recovery phase may be attributed to T-cell activation and differentiation.

## 1 Introduction

Severe fever with thrombocytopenia syndrome (SFTS) is an emerging infectious disease caused by a SFTS virus (SFTSV), which is classified into the genus *Phlebovirus* and family *Bunyaviridae*.^[1]^ The SFTSV is a tick-borne virus, and humans and animals are infected by bites from infected ticks.^[2]^ The fatality rate for STFS ranges from 12% to 30%.^[3]^ Severe cases of SFTS can present hemophagocytic lymphohistiocytosis (HLH) associated with a cytokine storm.^[4]^

While cytokine profiles in SFTS patients have been reported in several case series,^[5–9]^ few reports describe the dynamic changes in cytokine profile during the clinical course.^[10–12]^ Here, we have serially evaluated the changes in the SFTS viral load and serum cytokine and chemokine profiles during the course of the disease in a patient with SFTS.

## 2 Case report

**Figure 1:**
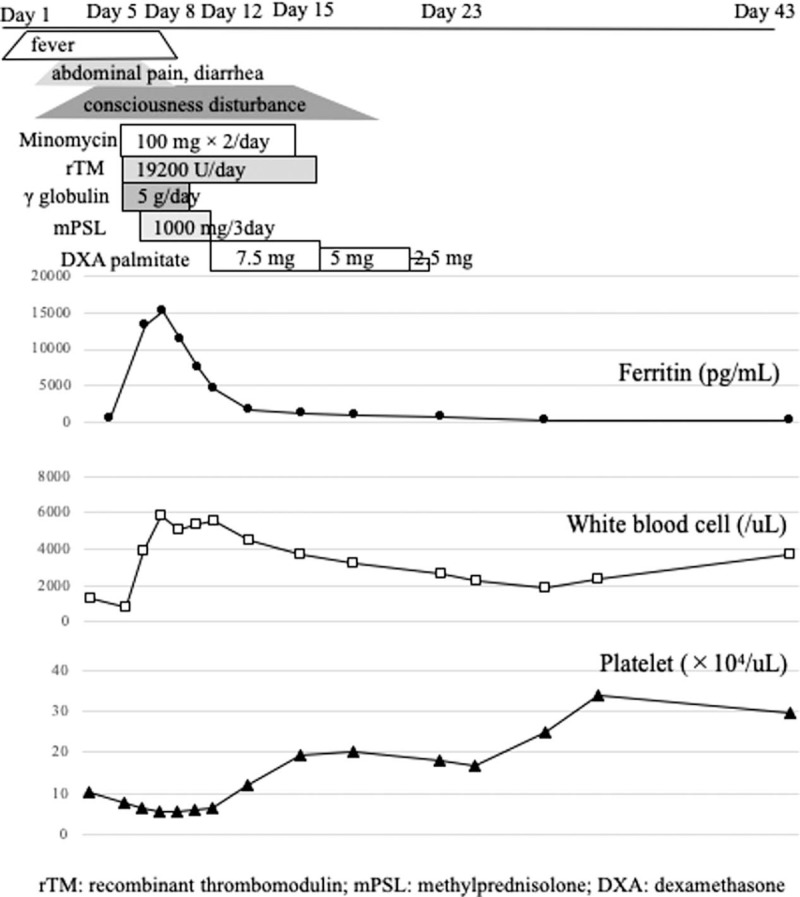
Clinical course.

**Figure 2:**
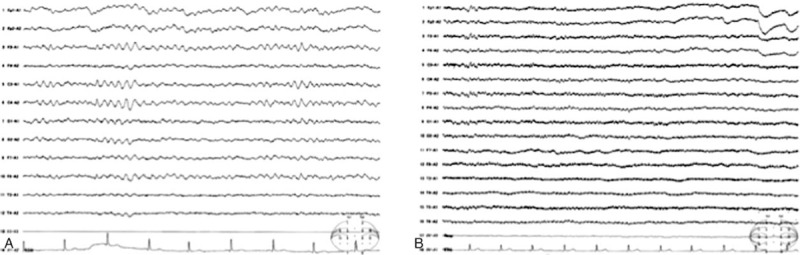
EEG on day 10 of illness (A) and on day 29 (B). (A) EEG shows slow waves such as *θ* and *δ*. (B) Slow waves are not observed. EEG = Electroencephalogram.

In 2017, a 63-year-old Japanese woman suffering for 2 days of a high-grade fever and general malaise was transported to our hospital. At presentation, her vital signs were as follows; blood pressure, 165/82 mm Hg; pulse rate, 83 beats/min; and temperature, 40.3°C and a detailed physical examination revealed tenderness and swelling of right inguinal lymph node. Her medical history was remarkable for type 2 diabetes mellitus. She did not have a stab wound by mites. Laboratory data revealed depressed white blood cell (WBC; 1260/μL) and platelet (Plt; 10.2 × 10^4^/μL) counts, and slightly elevated levels of aspartate aminotransferase (75 IU/L), alanine aminotransferase (58 IU/L), lactate dehydrogenase (245 IU/L), ferritin (441.1 ng/mL), and C-reactive protein (0.26 mg/dL). The finding of computed tomography (CT) scans was remarkable only for right inguinal lymph node swelling. The clinical course of the patient is depicted in Figure 1. After admission, her symptoms worsened with the development of abdominal pain, diarrhea, and headache. On day 5 of her illness, the profile of the laboratory data also worsened to WBC and Plt counts of 860/μL and 7.8 × 10^4^/μL, respectively, with impaired consciousness and a Glasgow coma scale of E3V3M6. A head CT detected no abnormal findings, but cerebrospinal fluid (CSF) protein levels were mildly elevated (63 mg/dL) with no increase in the cell counts. An electroencephalogram (EEG) revealed diffuse slow waves such as the *θ* and the *δ* waves (Fig. 2A). A bone marrow aspiration showed slightly hemophagocytosis but no abnormal cells. Based on the above symptoms, we asked the public health authority to test for SFTSV in the patient's serum. The patient tested positive for SFTSV RNA by polymerase chain reaction (PCR) and negative for *Orientia tsutsugamushi* and *Rickettsia japonica.* Thus, a diagnosis of SFTS was established on day 6. On day 7 of illness, the serum ferritin levels were markedly elevated to 15,070 ng/mL, and as there was concern about complications with HLH and disseminated intravascular coagulation, the patient was treated with intravenous dexamethasone palmitate (7.5 mg/d), steroid pulse therapy (methylprednisolone 1 g/d for 3 days), as well as intravenous recombinant thrombomodulin 19200 U/d for 7 days, minomycin 100 mg twice daily for 6 days, and gamma globulin 5 g/d for 3 days. Her symptoms and laboratory profile improved with this treatment and the impairment in consciousness recovered gradually. An EEG on day 29 showed normal findings with disappearance of the slow waves (Fig. 2B). After her consciousness recovered, it was revealed that she had exterminated mites in an asparagus farm 2 weeks before the hospitalization. The patient was discharged on day 32 without any neurological sequelae.

We estimated the serum SFTS viral load by RT-PCR and the serum cytokine and chemokine levels by multiplex PCR assays at days 8, 10, 12, 15, 23, 29, and 43 of the illness (Table 1). SFTSV was detected on day 8 (5.86 log^10^ RNA copies/mL), but not after day 10. The elevation in serum cytokine and chemokine levels could be divided into 2 patterns, namely, during the acute phase and during the recovery phase. The cytokines and chemokines elevated in the acute phase (day 8) were interleukin (IL)-1α, IL1-receptor antagonist, IL-6, IL-10, IL-15, interferon (IFN)-α2, IFN-γ, interferon-γ-induced protein (IP)-10, tumor necrosis factor (TNF)-α, granulocyte-colony stimulating factor, macrophage inflammatory protein (MIP)-1β, and fractalkine, while those elevated in the recovery phase (from days 23–43) included IL-1β, IL-8, IL-12p40, IL-17, granulocyte macrophage colony-stimulating factor, growth-regulated oncogene, monocyte chemoattractant protein (MCP)-1, MIP-1α, macrophage-derived chemokine, vascular endothelial growth factor (VEGF), epidermal growth factor, eotaxin, and soluble CD40 Ligand.

**Table 1:**
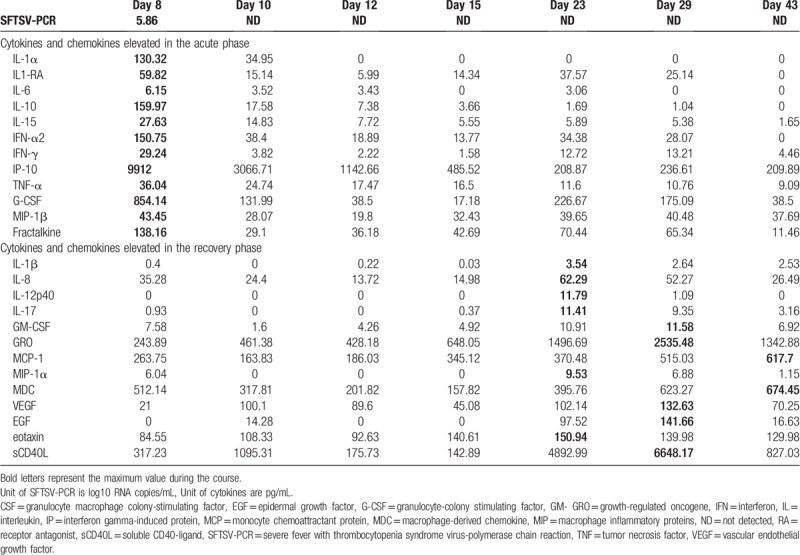
Severe fever with thrombocytopenia syndrome viral load and serum cytokine and chemokine levels in the present case.

## 3 Discussion

We describe the case of a 63-year-old woman suffering from SFTS and impaired consciousness and the dynamic changes in serum cytokine and chemokine levels. Supportive therapy, including steroid pulse therapy and recombinant thrombomodulin, improved her symptoms and EEG findings. To the best of our knowledge, this is first case report of a Japanese patient with SFTS that has evaluated viral load and changes in serum cytokine and chemokine profile.

Encephalitis or encephalopathy occurs in 19.1% of patients with SFTS,^[13]^ and encephalitis is seen in severe and fatal cases. Although a few cases of pleocytosis and elevated protein level in CSF have been reported, 75% of the patients test positive for STFS virus in the CSF.^[14]^ Additionally, the MCP-1 and IL-8 levels in the CSF are significantly higher than those in the serum. The common EEG finding in encephalopathy is diffuse slow-wave, focal or lateralized slow-wave, or focal seizure discharge.^[15]^ In our case, the SFTS viral load and the cytokine profile of the CSF were not analyzed, but based on EEG findings, the impaired consciousness was assumed to be related to encephalopathy associated with SFTS infection.

Patients with high viral load at hospitalization have poor prognosis. Zhang et al have reported that 8 of 10 patients with serum viral loads of ≥10^5^ copies/mL at admission died, but that all patients with viral loads of <10^5^ copies/mL survived.^[16]^ Kwon et al have reported that the SFTS viral RNA was persistently high in fatal cases, but that in survivors, the virus could not be detected at 2 to 3 weeks.^[12]^ In our case, the viral load was 5.86 log^10^ copies/mL on day 8 of illness, but its level decreased by day 10, and was accompanied by an improvement in the clinical symptoms. The pathological features of SFTS patients were necrotizing lymphadenitis in lymph node and hemophagocytosis in bone marrow, spleen, and lymph node.^[17]^ Immunohistochemical staining of lymph node showed that positive staining for the SFTSV-nucleoprotein (SFTSV-NP) was detected in the cytoplasm of atypical lymphoid cells.^[17]^ Because SFTSV-NP positive cells were most frequently observed in lymph node, it is speculated that SFTSV infects and proliferates primarily in lymph node.

Several case series have reported cytokine and chemokine profiles in SFTS patients, especially from China. Table 2 presents a literature review of the cytokine and chemokine profiles in 2 distinct phases:

(1) the acute phase within 14 days of illness,
(2) the recovery phase after 15 days of illness.

**Table 2:**
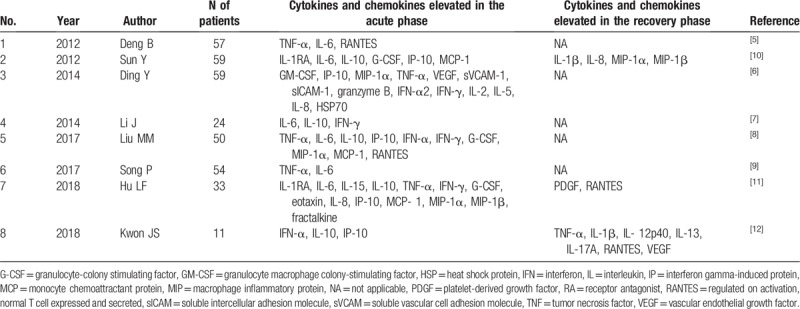
A review of previous reports that have evaluated cytokine and chemokine profiles in severe fever with thrombocytopenia syndrome patients.

It is evident from this table that cytokines and chemokines such as IL-6, IL-10, IP-10, IFN-α, IFN-γ, TNF-α, MCP-1, and MIP-1α were frequently elevated in the acute phase of SFTS, and the chemokine and cytokine profile of our patient is consistent with these published results. Additionally, several reports have demonstrated that IL-6, IL-10, and IP-10 were commonly elevated in fatal cases compared to those in who survive.^[6,7,10,11]^ Kwon et al have also demonstrated that IFN-α, IFN-γ, IL-10, MCP-1, chemokine (C-X-C motif) ligand 8 (IL-8), and IP-10 levels significantly correlate with viral load.^[12]^ IFN-α, which is type-1 IFN, contributes to balancing viral load with immune response.^[18]^ Despite exerting an antiviral effect by inhibiting virus replication, IFN-1 signaling promotes mortality through induction of aberrant inflammatory responses during the acute phase. As not only IFN-α but also other pro-inflammatory cytokines were elevated in our patient, these results reflect cytokine storms and disease severity during the acute phase of SFTS.

In contrast, elevation of cytokines and chemokines such as IL-1β, IL-8, MIP-1α, MIP-1β, platelet-derived growth factor (PDGF), regulated on activation, normal T cell expressed and secreted (RANTES), TNF-α, IL-1β, IL-12p40, IL-13, IL-17A, and VEGF have been reported to occur during the recovery phase,^[10–12]^ and our observations in this case similar with those in published reports. Weng et al reported that CD3+ CD4+ T cells were significantly decreased in acute phase and increased in recovery phase in peripheral blood lymphocyte subsets analysis.^[19]^ Hu et al have analyzed plasma concentrations of PDGF and RANTES, which are released by activated Plts, and state that serum levels were positively correlated with Plt counts.^[11]^ RANTES plays a role in recruiting and activating T cells. Kwon et al also suggest that the elevation in IL-13 and IL-17A may contribute to shaping T-cell immunity toward Th2 and Th17 responses.^[12]^ These results indicate that cytokines and chemokines elevated in the recovery phase are associated with recovery of blood cells and T-cell activation and differentiation.

Although the pathology of SFTS remains unclear, the cytokine storm induced by SFTS infection is associated with poor prognosis and it is thus essential to regulate the cytokine storm. Immunosuppressive therapy such as steroid pulse therapy and plasma apheresis might be effective in reducing the serum level of cytokines. SFTS viral load is also associated with prognosis and it is important to regulate viral replication. A clinical trial to evaluate the efficacy of favipiravir in SFTS patients is ongoing in Japan.^[20]^ It is important to ensure an improvement in prognosis by introducing these treatments at an early stage during SFTS infection.

## 4 Conclusions

We describe the case of a SFTS patient with encephalopathy along with serial changes in serum cytokine and chemokine profiles. The serum pro-inflammatory cytokine and chemokine levels and SFTS viral load were elevated in the acute phase; these improved immediately along with clinical symptoms. On the other hand, several cytokines and chemokines were elevated in the recovery phase along with blood cells recovery. Analysis of the changes in the cytokine profile and viral load in SFTS patients is important for understanding the pathology of SFTS infection and for improving prognosis.

## Author contributions

**Conceptualization:** Keita Fujikawa, Toshihisa Uchida, Momoko Okamoto, Yushiro Endo, Tomo Mihara, Akira Kondo, Akinari Mizokami.

**Data curation:** Keita Fujikawa, Takahide Honda.

**Investigation:** Tomohiro Koga, Satoshi Shimada, Daisuke Hayasaka, Kouichi Morita.

**Supervision:** Atsushi Kawakami.

**Writing – original draft:** Keita Fujikawa.

**Writing – review and editing:** Tomohiro Koga.
